# The impact of peri-interventional factors on pain reduction in glenohumeral corticosteroid injections

**DOI:** 10.1186/s12891-026-09754-5

**Published:** 2026-03-23

**Authors:** Karen Hübel, Susanne Bensler, Dusan Pisarcik, Jakob Heimer, Fabian Nicolas Jud

**Affiliations:** 1 Department of Medical Services, Institute of Radiology, Kantonsspital Baden, Baden, CH- 5404 Switzerland; 2 Loonstrasse 11C, Niederrohrdorf, 5443 Switzerland

**Keywords:** Glenohumeral injection, Shoulder joint, Corticosteroids, Sex differences, Patient education, Physician experience

## Abstract

**Background:**

Glenohumeral corticosteroid injections are widely used to treat shoulder pain, yet the influence of physician-related factors and patient education on clinical outcome remains unclear. This study evaluated the impact of physician sex, physician experience, and the extent of patient education on pain reduction following image-guided injections.

**Methods:**

In this prospective, single-centre observational cohort study, 193 patients undergoing fluoroscopy-guided glenohumeral corticosteroid injections were included. Pain intensity was assessed using a 10-point visual analogue scale (VAS) at baseline, 30 min, one week, and one month after the intervention. Pain outcomes were analysed using a linear mixed-effects model.

**Results:**

Pain scores decreased significantly at all follow-up time points compared with baseline (*p* < 0.001). No significant differences in pain reduction were observed with respect to patient education, physician sex, or physician experience (all *p* > 0.05).

**Conclusion:**

Fluoroscopy-guided glenohumeral corticosteroid injections provide effective short- and mid-term pain relief. The findings suggest that provider characteristics and the extent of patient education appear to have limited influence on patient-reported pain outcomes within this standardised procedural setting.

**Supplementary Information:**

The online version contains supplementary material available at 10.1186/s12891-026-09754-5.

## Highlights

Fluoroscopy-guided glenohumeral corticosteroid injections reduce shoulder pain significantly.

Patient education level does not significantly affect pain outcomes.

Radiologist sex and experience are not significantly associated with post-injection pain levels.

**Supplementary Information:**

The online version contains supplementary material available at 10.1186/s12891-026-09754-5.

## Introduction

Shoulder pain is considered to be the third most common musculoskeletal complaint after back and knee pain [1, 2]. Common causes include rotator cuff disorders, glenohumeral joint disorders, acromioclavicular joint disorders and referred neck pain [3, 4]. Initial treatment is usually conservative, with surgery reserved for a minority of cases. Intra-articular corticosteroid injections have been shown to significantly reduce pain in various conditions, both as monotherapy and in combination with conservative therapies [5, 6]. Depending on the underlying shoulder pathology, corticosteroid injections can be administered to different compartments of the shoulder [7], with the glenohumeral joint space being the most commonly targeted site. Prior studies have highlighted the importance of procedural factors in shoulder interventions and their potential influence on pain outcomes, particularly in the context of image-guided intra-articular injections [8]. As clinical outcome may be influenced by peri-interventional factors, such as the sex and experience of the performing physician [9], or the extent of the patient education prior to the procedure, the aim of this study was to investigate the impact of these variables on pain reduction following fluoroscopy-guided corticosteroid infiltration of the glenohumeral joint.

## Methods

### Study design

This prospective, single-centre observational cohort study was approved by the Ethics Committee Northwestern and Central Switzerland (EKNZ, ID: 2020 − 01891, 1 February 2021) and conducted in accordance with the Declaration of Helsinki. Written informed consent was obtained from all participants prior to the procedure for the use and publication of anonymised data.

From April 2021 to October 2024, a total of 198 patients were identified by a database search of our hospital information systems for patients referred to our radiology department for therapeutic or diagnostic fluoroscopy-guided glenohumeral corticosteroid injections. For patients with repeated referrals for infiltration, each injection was recorded as a separate observation in the dataset. 5 patients were excluded: 3 did not return the outcome questionnaire and could not be contacted, 1 withdrew consent, and 1 received a second glenohumeral injection 2 weeks after the first.

Finally, a total of 193 patients were included in the study (96 female, 97 male, mean age 56.4, age range 18–88). There were no exclusion criteria regarding the underlying cause of the shoulder pain.

Patients received one of two pre-procedural educational formats.

Format A (detailed; *n* = 96, treatment group) included all information provided in Format B and additionally incorporated illustrative material and a more detailed explanation of the procedure. These consisted of example images of a fluoroscopy-guided glenohumeral injection as well as a schematic of the shoulder skeleton demonstrating the injection. In addition, fluoroscopic guidance and radiation exposure were explained. This counselling was delivered verbally with visual support and typically required approximately 5 min.

Format B (standardised; *n* = 97, control group) consisted of a concise verbal explanation covering the essential procedural steps and main risks without the use of illustrative material. This included a brief description of the injection procedure and discussion of potential complications such as infection, bleeding, and allergic reactions to the injected medications, including routine inquiry about known allergies. This counselling typically required approximately 2–3 min.

The full educational materials used in both study groups are provided in the Supplementary Information (Supplementary Material 1).

Printed information sheets were mixed by the radiology team prior to clinical use and stored in a common stack. The treating physician selected the top sheet from this stack as part of routine clinical workflow and provided it to the patient. Treating physicians were unaware of which version was provided at the time of selection. No formal random sequence generation or allocation concealment was performed.

The performing radiologists were divided into two groups: experienced (more than 2 years of practice, *n* = 5) and inexperienced (less than 2 years of practice, *n* = 8). In total, 13 physicians performed the injections. The number of injections per physician ranged from 3 to 38 (median 14).

### Injection technique

All injections were performed by radiologists at our institution, with experience ranging from 6 months of residency to 10 years of specialised musculoskeletal practice.

**Fig. 1:**
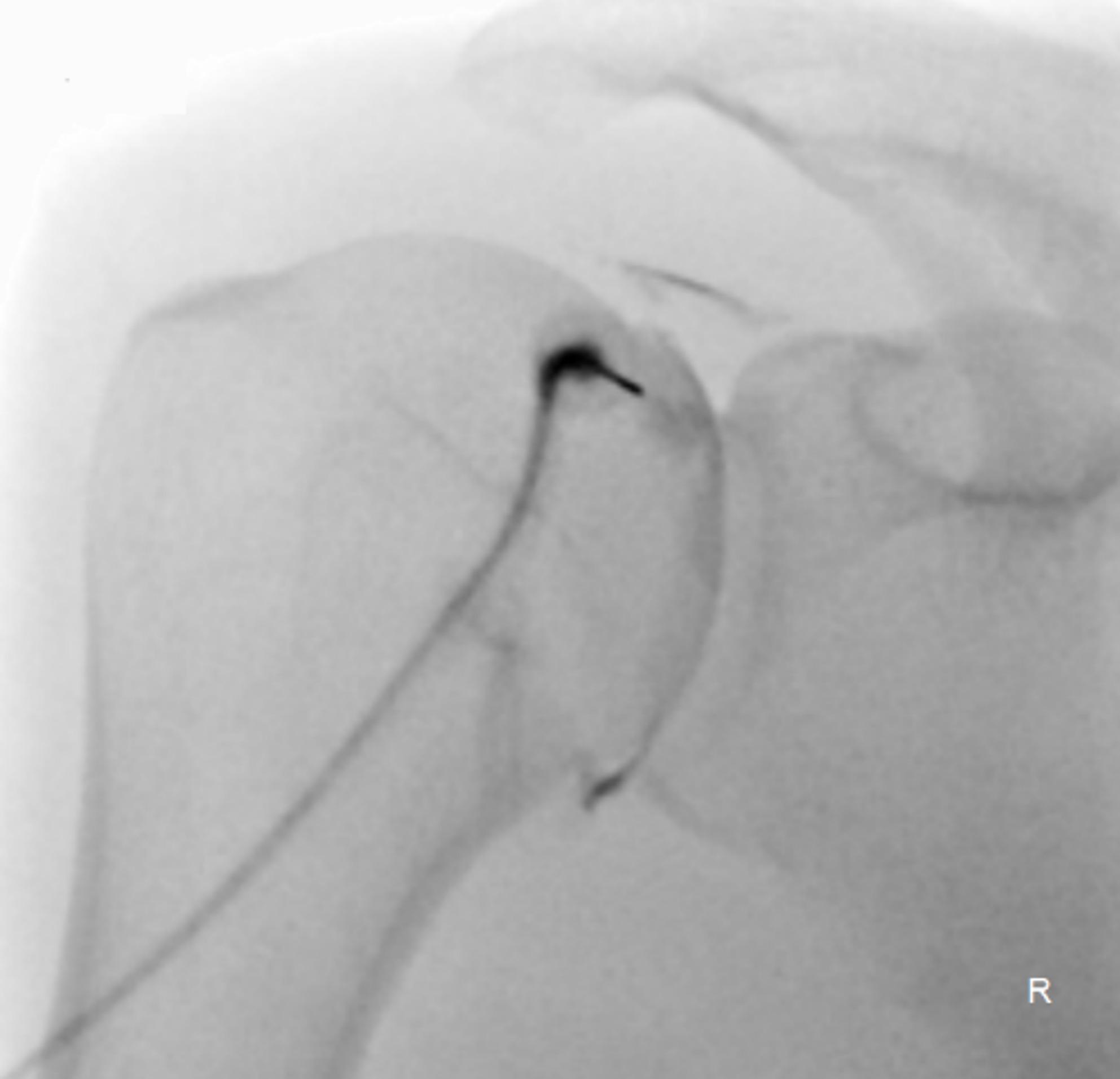
Fluoroscopy-guided glenohumeral injection. Correct needle positioning at the right upper medial humeral head is demonstrated, along with appropriate intra-articular contrast distribution within the glenohumeral joint space

Prior to the injection, a radiologic technician prepared all sterile materials and medications. A fully digital fluoroscopy system (Artis Zee, multi-purpose system, Siemens Healthcare GmbH, Erlangen) was used for image guidance. All patients were placed in supine position with elevation of the targeted shoulder at approximately 45 degrees. Under sterile conditions and fluoroscopic guidance, the radiologist advanced a 22-gauge needle onto the superior medial portion of the humeral head. Correct intra-articular needle placement was confirmed under fluoroscopy by injecting 1 ml of contrast medium (Iopamiro 200 or 300, Bracco Suisse SA, Manno, Switzerland. Figure 1). After confirmation, 1 ml corticosteroid suspension (Kenacort, 40 mg/ml, Dermapharm AG, Hünenberg, Switzerland) and 8–10 ml of local anaesthetic (Rapidocain 10 mg/ml, Sintetica, Mendrisio, Switzerland) were administered. Finally, the needle was removed and a sterile adhesive dressing was applied.

### Pain measurement

Prior to the procedure, all patients were asked to rate their pain on a visual analogue scale (VAS), ranging from 0 (“no pain”) to 10 (“worst imaginable pain”). The same assessment was repeated 30 min after the injection. Patients were provided with VAS forms to complete at home at one week and one month after the injection. A prepaid return envelope was included to facilitate the return of completed questionnaires after one month.

### Statistical analysis

Baseline characteristics between the control and treatment groups were compared using Wilcoxon rank-sum tests for continuous variables (patient age, baseline pain VAS) and Pearson’s Chi-squared tests for categorical variables (patient sex, doctor experience, doctor sex). Continuous variables are presented as mean (standard deviation, SD).

To analyse the change in pain scores (VAS) over time and between groups, a linear mixed-effects model was fitted. The model included fixed effects for time point (Baseline, After Intervention, One Week, One Month), treatment group (Control, Treatment), and their interaction. Patient sex, patient age, doctor experience (Experienced, Inexperienced), and doctor sex were included as fixed covariates. Random intercepts were included for both patient (subject ID) and doctor (doctor ID) to account for repeated measures within patients and potential clustering by doctor. Variance components for random effects were estimated.

Mixed-effects models were estimated using maximum likelihood, which allows inclusion of observations with incomplete outcome data under a missing-at-random assumption. Response mode (postal vs. telephone follow-up) was examined as a potential covariate and showed no meaningful association with pain scores; inclusion of response mode in the model did not materially alter the estimated effects. Model assumptions were assessed by visual inspection of residual plots (normality and homoscedasticity) and were deemed acceptable. No imputation or interpolation of missing VAS values was performed. Estimated marginal means were calculated for the time-by-group interaction. Coefficients for fixed effects are reported with 95% confidence intervals (CIs). A *p*-value < 0.05 was considered statistically significant. Analyses were performed using R statistical software (Version 4.4.1).

## Results

A total of 193 patients were included in the final analysis, with 97 allocated to the control group (standard education) and 96 to the treatment group (detailed education). 156 patients returned the VAS form by postal service, 37 patients who did not return their questionnaire or had incomplete entries were contacted by telephone. If follow-up information could not be obtained for a specific time point, that time point was recorded as missing, while other available measurements for the same patient were retained in the analysis. 7 patients underwent two separate injections and therefore contributed two observations to the dataset. A sensitivity analysis excluding second injections (*n* = 7) yielded materially unchanged results (see Supplementary Material 2).

Baseline characteristics were well-balanced between the two groups, with no statistically significant differences observed in patient sex, mean age, baseline pain VAS score, radiologist experience, or radiologist sex distribution (Table 1).

**Table 1:** Baseline characteristics of the study population

Variable	Control Group *N* = 97^*1*^	Treatment Group *N* = 96^*1*^	*P*-value^2^	Overall *N* = 193^*1*^
Patient Sex			0.72	
Male	50 / 97 (52%)	47 / 96 (49%)		97 / 193 (50%)
Female	47 / 97 (48%)	49 / 96 (51%)		96 / 193 (50%)
Patient Age	56.3 (14.6)	56.5 (14.4)	0.75	56.4 (14.5)
Baseline Pain (VAS)	5.8 (2.3)	6.1 (2.3)	0.46	6.0 (2.3)
Doctor Experience			0.83	
Experienced	47 / 97 (48%)	45 / 96 (47%)		92 / 193 (48%)
Inexperienced	50 / 97 (52%)	51 / 96 (53%)		101 / 193 (52%)
Doctor Sex			0.28	
Male	53 / 97 (55%)	45 / 96 (47%)		98 / 193 (51%)
Female	44 / 97 (45%)	51 / 96 (53%)		95 / 193 (49%)

^*1*^ Mean (SD) for continuous variables; n / N (%) for categorical variables

^*2*^ Pearson’s Chi-squared test; Wilcoxon rank sum test

The linear mixed-effects model revealed a significant main effect of time on pain scores (Table 2). Compared to baseline, pain scores were significantly lower after intervention (β = -2.0, 95% CI [-2.5, -1.5], *p* < 0.001), at one week (β = -3.1, 95% CI [-3.5, -2.6], *p* < 0.001), and at one month (β = -3.0, 95% CI [-3.4, -2.5], *p* < 0.001). Further post-hoc comparisons (Table 3) indicated that pain scores at ‘One Week’ were significantly lower than at ‘After Intervention’ (mean difference = -0.93, 95% CI [-1.28, -0.59], *p* < 0.001), and similarly, pain scores at ‘One Month’ were significantly lower than at ‘After Intervention’ (mean difference = -0.94, 95% CI [-1.28, -0.59], *p* < 0.001). There was no significant difference in pain scores between ‘One Week’ and ‘One Month’ (mean difference = 0.01, 95% CI [-0.34, 0.35], *p* = 1.000).

**Table 2:** Mixed-effects model coefficients for pain score (VAS)

Predictor	Coefficient	95% CI^1^	*p*-value
Time Point			
Baseline	—	—	
After Intervention	-2.0	-2.5, -1.5	< 0.001
One Week	-3.1	-3.5, -2.6	< 0.001
One Month	-3.0	-3.4, -2.5	< 0.001
Treatment Group			
Control Group	—	—	
Treatment Group	0.30	-0.39, 0.98	0.394
Patient Sex			
Male	—	—	
Female	0.71	0.17, 1.3	0.010
Doctor Experience			
Experienced	—	—	
Inexperienced	-0.77	-1.6, 0.09	0.067
Doctor Sex			
Male	—	—	
Female	0.06	-0.76, 0.89	0.838
Patient Age (years)	-0.02	-0.04, 0.00	0.081
Time × Group Interaction			
After Intervention * Treatment Group	-0.36	-1.1, 0.32	0.298
One Week * Treatment Group	-0.04	-0.73, 0.64	0.904
One Month * Treatment Group	-0.27	-0.96, 0.42	0.440

Reference levels: Group=’Control Group’, Time=’Baseline’, Patient Sex=’Male’, Doctor Experience=’Experienced’, Doctor Sex=’Male’. Coefficients represent change relative to reference category. Negative coefficients indicate greater pain reduction on the visual analogue scale (VAS)

^1^ *CI* Confidence Interval

**Table 3:** Post-hoc comparisons of pain scores across time points

Comparison	Estimate (Difference)	95% CI Lower	95% CI Upper	*P*-value
Baseline - After Intervention	2.15	1.81	2.49	0.000
Baseline - One Week	3.08	2.74	3.43	0.000
Baseline - One Month	3.09	2.75	3.43	0.000
After Intervention - One Week	0.93	0.59	1.28	0.000
After Intervention - One Month	0.94	0.59	1.28	0.000
One Week - One Month	0.01	-0.34	0.35	1.000

Estimates represent the difference in mean pain VAS. Tukey adjustment for *p*-values

**Fig. 2:**
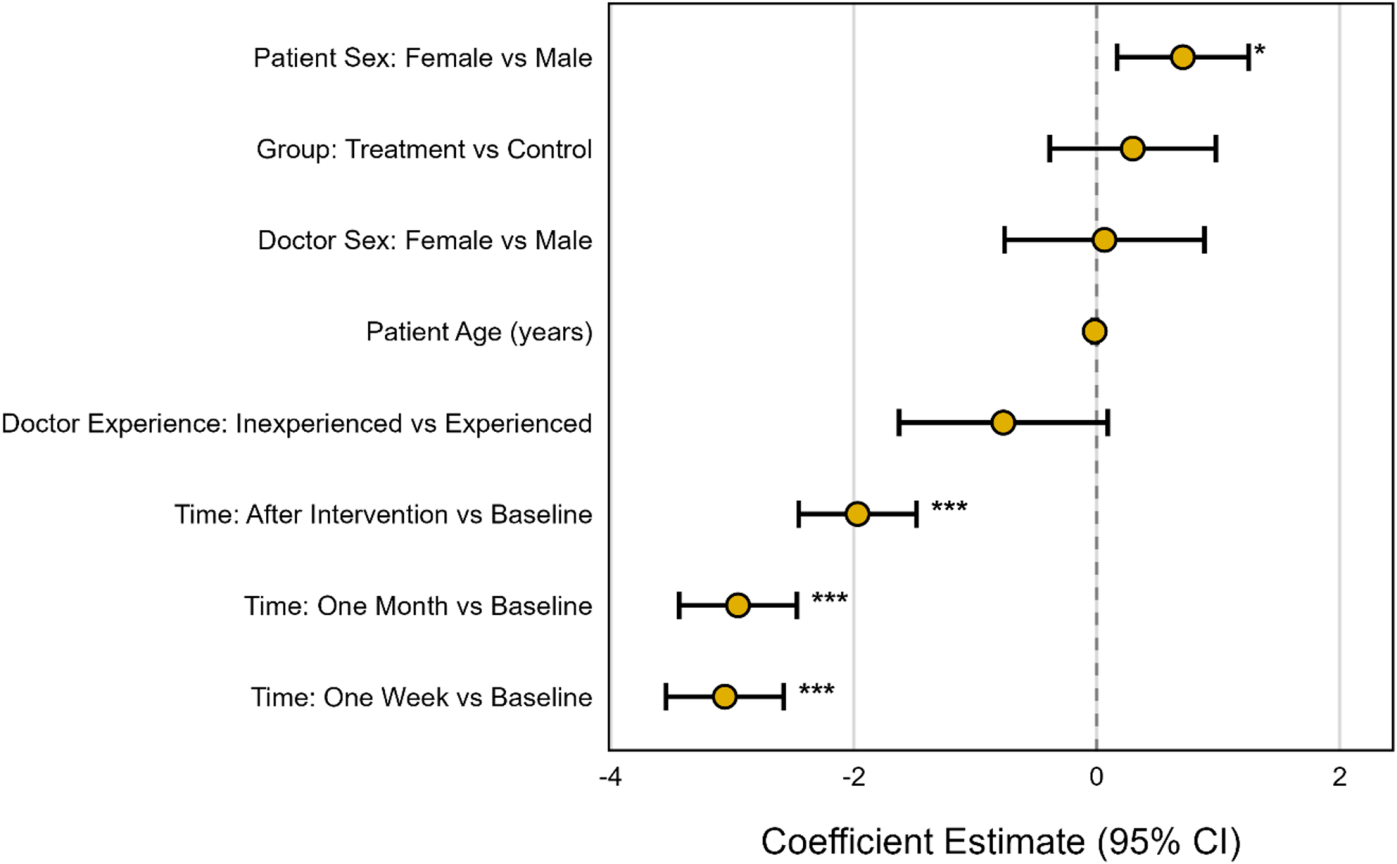
Forest plot of regression coefficients from the linear mixed-effects model. Points represent estimated coefficients (β) and horizontal lines indicate 95% confidence intervals for the fixed effects. Negative coefficients indicate greater pain reduction on the visual analogue scale (VAS). Significance levels are indicated by asterisks (* *p* < 0.05, ** *p* < 0.01, *** *p* < 0.001)

No significant main effect was found for the treatment group (Treatment vs. Control: β = 0.30, 95% CI [-0.39, 0.98], *p* = 0.394). Furthermore, the interaction between time and treatment group was not statistically significant at any follow-up time point (*p* > 0.29 for all interaction terms), indicating that the change in pain over time did not significantly differ between the treatment and control groups (Table 2, Fig. 2).

**Fig. 3:**
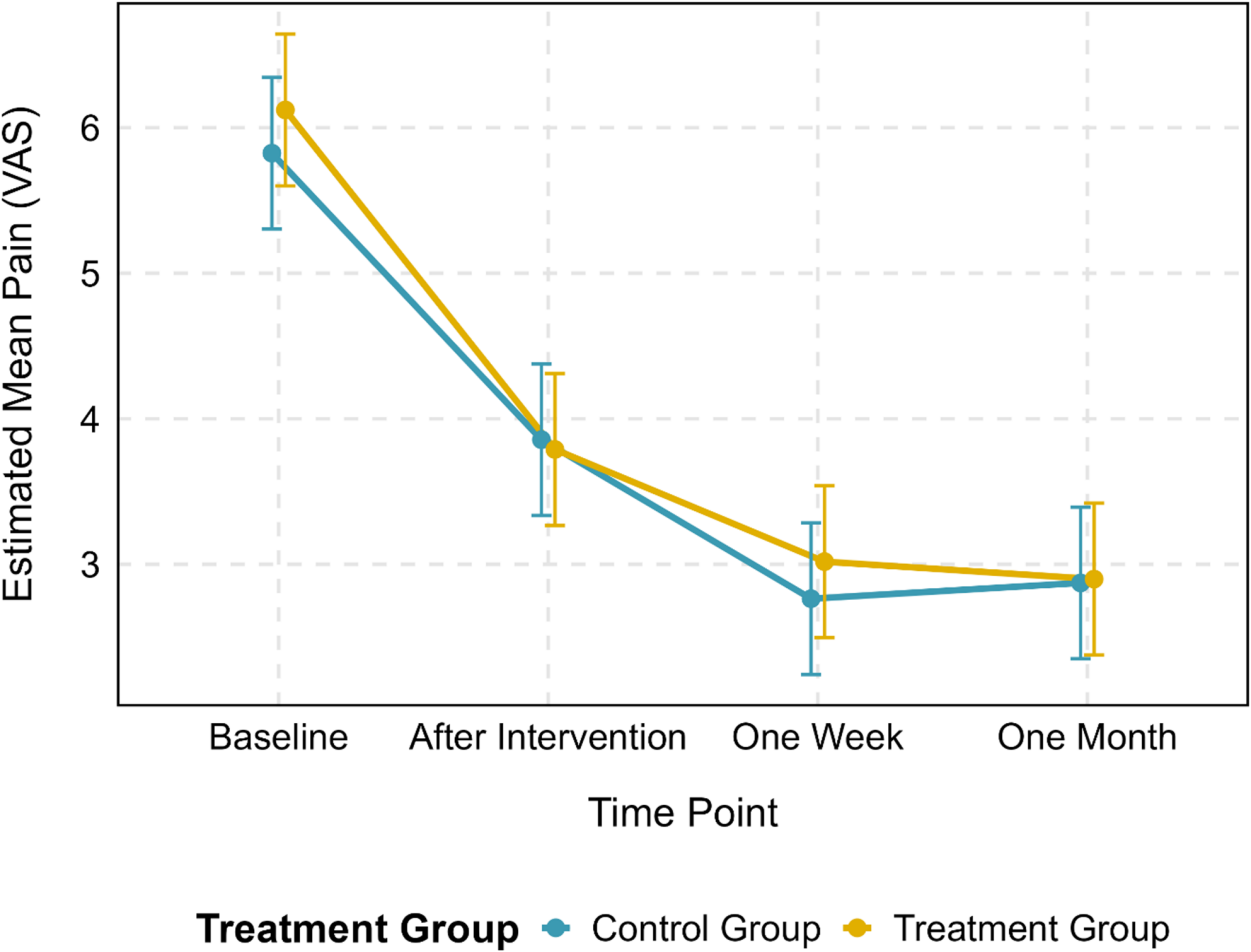
Interaction plot of pain score over time by treatment group. Estimated mean pain score (VAS) over time points, stratified by treatment group. Error bars represent 95% confidence intervals

Among the covariates, female patient sex was associated with significantly higher pain scores compared to male patients (β = 0.71, 95% CI [0.17, 1.3], *p* = 0.010). Trends towards lower pain scores were observed for patients treated by inexperienced doctors (β = -0.77, 95% CI [-1.6, 0.09], *p* = 0.067) and for younger patients (β = -0.02 per year, 95% CI [-0.04, 0.00], *p* = 0.081), although these did not reach statistical significance. The performing physician’s sex was not significantly associated with pain scores (*p* = 0.838) (Table 2, Fig. 3).

Analysis of variance components showed that patient-level variability accounted for the majority of variance in pain scores, whereas variability attributable to the performing physician was minimal. This indicates that patient-specific factors contribute substantially more to pain score variation than physician-related factors after adjustment for fixed effects.

## Discussion

This study aimed to assess whether patient education or the physician’s sex and experience affected pain levels after glenohumeral corticosteroid injection. The results demonstrated no statistically significant association between any of these factors and the observed treatment outcomes.

### Patient education

Although previous studies have examined the effect of patient education on pain levels in other interventional and surgical procedures [10, 11], its impact on glenohumeral joint injections has been insufficiently studied. In our study, no significant difference in outcomes was found between patients who received a standard consent form and those given a detailed education by the radiologist.

This may be related to the procedure’s simplicity and minimally invasive nature, which requires little patient cooperation or post-procedural compliance. As such, extensive pre-procedural education may offer limited additional clinical benefit in this setting, although small effects cannot be excluded.

In contrast, prior research suggests that patient-centred interaction during procedures such as using the patient’s name, maintaining eye contact, and expressing positive expectations can improve pain outcomes [12]. Enhancing physician patient interaction during the procedure may therefore be a more effective strategy than expanding pre-procedural education.

### Physician experience

We found no significant differences in pain relief between patients treated by experienced and inexperienced radiologists. This may also be due to the simplicity of the injection technique, which most residents can perform reliably after minimal supervision. Additionally, the use of contrast media under fluoroscopic guidance allows for dependable confirmation of intra-articular needle placement, even by less experienced physicians.

These findings are broadly consistent with other publications assessing provider experience under fluoroscopic guidance [13, 14]. Additionally, a study by Tobola et al. [15] compared the accuracy of blind injections performed by experienced and inexperienced clinicians using different approaches (anterior, posterior, and supraclavicular). While the anterior approach (which was also used in our study) was the most accurate, they found no significant advantage for the experienced orthopaedic surgeons compared to their less experienced colleagues. In fact, the less experienced group actually performed slightly better, although this was not statistically significant.

In contrast, Mattie et al. [16] reported statistically significant differences based on provider experience but found no clear benefit of the anterior approach. Both studies, however, emphasized the importance of image guidance to increase accuracy over blind techniques.

In this context, our findings suggest that with fluoroscopic guidance, provider experience was not significantly associated with short- or mid-term pain outcomes. This may have important clinical implications, suggesting that clinically meaningful differences between experience levels may be limited within a standardised, image-guided setting.

### Physician sex

An increasing number of studies explore differences in patient care between female and male physicians. Studies suggest that female physicians tend to practice more conservatively, offer more patient-centred care, and provide more preventive services, while male physicians may focus more on technical tasks such as history taking and physical exams [17–19]. In diagnostic radiology, female physicians tend to have a narrower scope of practice but make fewer mistakes [20].

However, no prior studies have assessed the impact of physician sex on outcomes after joint infiltrations. In related fields, Thöni et al. reported that female chiropractors achieved equal or better outcomes in treating neck pain and especially in acute lower back pain [21, 22].

Our findings did not show a statistically significant association between provider sex and treatment outcomes in this cohort.

### Patient sex

In our cohort, female patients reported significantly higher pain scores. Sex-related differences in pain perception have been described in previous studies and may reflect a combination of biological, hormonal, and psychosocial factors influencing pain processing and reporting [23, 24]. However, the present study was not designed to investigate mechanisms underlying these differences, and this finding should therefore be interpreted cautiously. From a clinical perspective, awareness of potential sex-related differences in pain perception may be relevant when counselling patients and interpreting patient-reported outcomes following musculoskeletal interventions. Future studies may further explore whether such differences influence treatment expectations, pain reporting, or response to image-guided interventions.

### Limitations

This study has several limitations. First, it was conducted at a single-centre, which may not be applicable to other institutions. Second, early pain reduction at 30 min is likely influenced predominantly by the local anaesthetic component of the injection and should therefore be interpreted with caution when attributing effects to corticosteroid therapy. In addition, the relatively large volume of local anaesthetic may have reduced sensitivity to detect small differences related to the educational intervention. Third, follow-up was limited to one month, preventing assessment of long‑term outcomes or delayed treatment effects [25, 26]. Furthermore, 37 patients did not return the mailed questionnaire and were contacted by phone, which may have resulted in more favourable responses, as suggested by Lechmann et al. [27].

In addition, a small number of patients underwent repeated injections, which could introduce non-independence of observations, although sensitivity analysis showed no material change in the results. Underlying shoulder pathologies were not analysed separately and may influence pain trajectories. A further limitation is that the study was not prospectively registered as a clinical trial. However, the study was designed as an observational cohort study without interventional assignment, and all procedures were performed as part of routine clinical care. Finally, the study may have been underpowered to detect small subgroup effects related to physician characteristics or patient education.

## Conclusions

Fluoroscopy-guided corticosteroid injection of the glenohumeral joint is an effective and established treatment option for shoulder pain, with significant pain reduction observed at all follow-up intervals. In this study, neither the extent of patient education nor the physician’s level of experience or sex showed a statistically significant association with patient-reported pain outcomes. These findings suggest that, within the context of a standardised and image-guided procedure, clinically meaningful differences between providers may be limited; however, small effects cannot be fully excluded. Overall, the findings support the robustness of this commonly used intervention across routine clinical settings.

## Supplementary Information

Supplementary Material 1.

Supplementary Material 2.

## Data Availability

The datasets used and/or analysed during the current study are available from the corresponding author on reasonable request.

## Abbreviations

SD: Standard deviation
VAS: Visual analogue scale
