# Supplementary material for: The impact of peri-interventional factors on pain reduction in glenohumeral corticosteroid injections
Source: BMC Musculoskelet Disord. 2026 Mar 23;27:352. doi: 10.1186/s12891-026-09754-5 (PMC13107596; doi:10.1186/s12891-026-09754-5)
Supplement: Supplementary file 1 — Supplementary Material 1. [file 12891_2026_9754_MOESM1_ESM.pdf]

Supplementary Material A. Detailed illustrated pre-procedural patient education

This document contains the detailed illustrated patient education material (Format A) used prior to fluoroscopy-guided glenohumeral joint injection. The original material was used in routine clinical practice; the present version is an English translation provided for transparency and reproducibility.

Patient Information: Fluoroscopy-guided glenohumeral joint injection

Purpose of the procedure

You have been scheduled for a fluoroscopy-guided injection of the glenohumeral (shoulder) joint. The aim of this procedure is to deliver medication (local anaesthetic and corticosteroid) directly into the joint space in order to reduce pain and inflammation.

Procedure

The injection is performed under imaging guidance using fluoroscopy (X-ray). After sterile skin preparation, a thin needle is advanced into the shoulder joint under imaging control. A small amount of contrast medium may be injected to confirm correct intra-articular needle position. Subsequently, the therapeutic medication is administered into the joint.

Illustrations are used to explain the anatomical structures and the course of the injection to improve patient understanding.

Radiation exposure

The procedure is performed under fluoroscopic guidance and therefore involves a small amount of X-ray exposure. The radiation dose is kept as low as reasonably achievable and is generally considered low.

Possible risks and complications

As with any medical procedure, complications cannot be completely excluded, but serious adverse events are rare. Potential risks include:

- infection at the injection site
- bleeding or haematoma
- allergic reaction to the administered medications or contrast agent
- temporary increase in pain after the injection

All procedures are performed under sterile conditions to minimise risk.

After the procedure

Initial pain relief may occur due to the local anaesthetic shortly after the injection. The anti-inflammatory effect of the corticosteroid typically develops within several days. You may be advised to temporarily reduce strain on the treated shoulder.

If you experience unusual symptoms such as increasing pain, fever, or signs of infection, please contact your treating physician.

Supplementary Material A (*Original German patient information sheet*).

Durchleuchtung-gesteuerte Schultergelenkinfiltration

Sehr geehrte Patientin, sehr geehrter Patient

Bitte lesen Sie dieses Formular aufmerksam durch, damit Sie der zuständigen Ärztin/dem zuständigen Arzt gegebenenfalls zusätzliche Fragen stellen können.
Bitte teilen Sie es uns mit, falls bei Ihnen eine Blutverdünnung durchgeführt wird oder Sie unter Allergien leiden.

Art des Eingriffs

Ihre behandelnden Ärztinnen/Ärzte weisen Sie zur Punktion des glenohumeralen Gelenkes (= Schultergelenks) zu mit gezielter Verabreichung schmerzlindernder Medikamente in den Gelenkraum.

Verlauf des Eingriffs

Mit Hilfe eines Durchleuchtungsgeräts (im Prinzip Röntgengerät) wird eine Aufnahme des betreffenden Schultergelenkes angefertigt (Abb. 1), somit kann der Radiologie gezielt unter sterien Bedingungen eine feine Hohlnadel bis in den Gelenkraum unter Applikation des Betäubungsmittels vorführen und die Lage mit der Bildgebung kontrollieren (Abb. 2). Bei korrekter Nadellage wird zuerst ein Kontrastmittel unter Durchleuchtung appliziert und nach Bestätigung der korrekten Nadellage durch Ausbreitung des Kontrastmittels innerhalb des Schultergelenkraums (Abb. 3) wird ein Cortison-Präparat und anschliessend der Rest von dem Betäubungsmittel injiziert. Während der Applikation der Medikamente werden Sie in dem Schultergelenk höchstens ein Druckgefühl spüren.

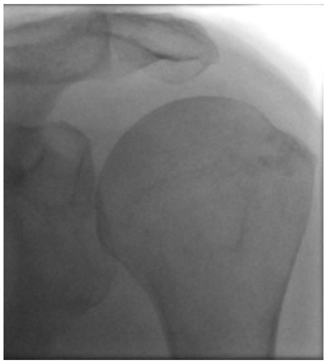
Abb. 1

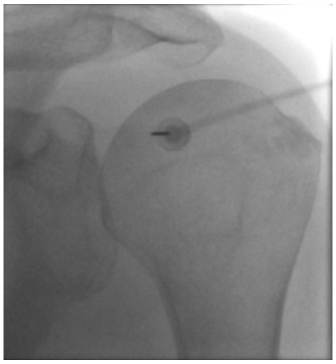
Abb. 2

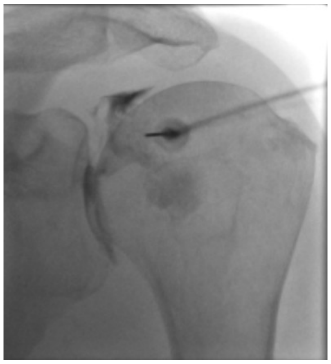
Abb. 3

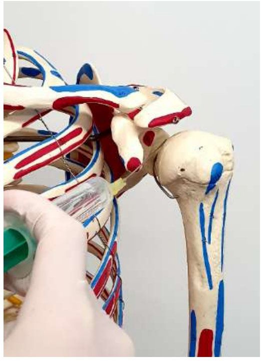
Abb. 4

Komplikationen

Risikofreiheit kann grundsätzlich bei keinem medizinischen Eingriff garantiert werden. Eine Gelenksinfektion ist unter sterilen Bedingungen mit grossflächiger Desinfektion der Punktionsstelle extrem selten. Eine Blutung oder Verletzung von benachbarten Strukturen ist ebenso extrem selten, wichtig ist, dass Sie das punktierte Schultergelenk kaum bewegen. Die Strahlenbelastung von dem Eingriff ist geringfügig. Bitte teilen Sie uns unbedingt mit, wenn Sie schwanger sein könnten oder an einer bekannten Überempfindlichkeit gegen örtliche Betäubungsmittel (Lokalanästhetika) leiden.

Weiteres Vorgehen

Zunächst wird das Ansprechen der Gelenkbeschwerden auf die lokalanästhetische Medikamentengabe abgewartet und Sie werden dazu, in der Regel eine halbe Stunde nach Medikamentenapplikation, befragt. Das Ansprechen auf das Cortison-Präparat ist erst nach 2 bis 3 Tagen zu erwarten. Bis dahin empfiehlt sich das betroffene Schultergelenk zu schonen.

Zur Beantwortung weiterer Fragen steht Ihnen die untersuchende Ärztin/der untersuchende Arzt sowie die Fachperson für medizinisch-technische Radiologie (MTRA) gerne zur Verfügung.

Supplementary Material B. Standard brief pre-procedural patient education

This document contains the standard brief patient education material (Format B) used prior to image-guided joint injection. The original material was used in routine clinical practice; the present version is an English translation provided for transparency and reproducibility.

Patient Information: Image-guided joint injection

You are scheduled to undergo an image-guided injection of a painful joint in order to administer medication directly into the joint space and help reduce inflammation and pain. This procedure is commonly used to relieve symptoms caused by inflammatory or degenerative joint conditions.

Procedure

The procedure is performed under imaging guidance (fluoroscopy). After disinfection of the skin, a thin needle is inserted into the joint space. The correct needle position is confirmed using imaging before medication is injected. The injected medication usually consists of a local anaesthetic for short-term pain relief and a corticosteroid to reduce inflammation.

Risks

No medical procedure can be considered entirely risk-free. Serious complications such as bleeding, joint infection, or injury to surrounding structures are extremely rare. Temporary discomfort or mild pain at the injection site may occur after the procedure.

Possible complications include:

- infection
- bleeding
- allergic reactions to the administered medications

If the injection is performed under fluoroscopic or CT guidance, a small amount of radiation exposure occurs. Please inform the medical staff if you might be pregnant or if you have a known allergy to local anaesthetic medications.

After the procedure

Pain relief from the local anaesthetic may occur shortly after the injection. The anti-inflammatory effect of the corticosteroid usually develops over the following days. Mild temporary discomfort at the injection site may occur.

If necessary, your treating physician will provide additional instructions regarding activity or follow-up.

Supplementary Material B (*Original German patient information sheet*).

Bildgebende gesteuerte Gelenkspunktion

Sehr geehrte Patientin, sehr geehrter Patient

Bitte lesen Sie dieses Formular aufmerksam durch, damit Sie der zuständigen Ärztin/dem zuständigen Arzt gegebenenfalls zusätzliche Fragen stellen können.
Bitte teilen Sie es uns mit, falls bei Ihnen eine Blutverdünnung durchgeführt wird oder Sie unter Allergien leiden.

Art des Eingriffs

Ihre behandelnden Ärztinnen/Ärzte weisen Sie zur Punktion des für Sie schmerzhaften Gelenkes (z.B. Hüftgelenk, Steissbeingelenk, Schultergelenk etc.) zu mit gezielter Verabreichung eines schmerzlindernden Medikamentes in den Gelenkraum.

Verlauf des Eingriffs

Mit Hilfe eines bildgebenden Verfahrens (Ultraschall, Durchleuchtung, Computertomographie [CT] etc.) werden Aufnahmen des betreffenden Gelenkes angefertigt. Anhand dieser Bilder entscheidet der Radiologe über den besten Zugang zum Gelenk. Unter sterilen Bedingungen wird eine feine Hohlnadel bis in den Gelenkraum vorgeführt und die Lage jeweils über die aktuelle Bildgebung kontrolliert. Bei korrekter Nadellage werden die Medikamente (Schmerzmittel zur örtlichen Betäubung sowie evt. Cortison-Präparat) in den Gelenkraum injiziert.

Komplikationen

Risikofreiheit kann grundsätzlich bei keinem medizinischen Eingriff garantiert werden. Ernsthafte Komplikationen wie Blutung, Gelenksinfektion oder Verletzung von benachbarten Organen sind extrem selten. Schmerzen nach der Punktion sind in der Regel höchstens geringfügig. Wird die Gelenkspunktion unter Durchleuchtung oder CT-Steuerung durchgeführt, besteht eine, wenn auch geringe Strahlenbelastung. Bitte teilen Sie uns unbedingt mit, wenn Sie schwanger sein könnten oder an einer bekannten Überempfindlichkeit gegen örtliche Betäubungsmittel (Lokalanästhetika) leiden.

Weiteres Vorgehen

Zunächst wird das Ansprechen der Gelenkbeschwerden auf die lokalanästhetische Medikamentengabe abgewartet und Sie werden dazu, in der Regel eine halbe Stunde nach Medikamentenapplikation, befragt. Das weitere Vorgehen wird dann die zuweisende Ärztin/der zuweisende Arzt mit Ihnen besprechen.

Zur Beantwortung weiterer Fragen steht Ihnen die untersuchende Ärztin/der untersuchende Arzt sowie die Fachperson für medizinisch-technische Radiologie (MTRA) gerne zur Verfügung.

Ich wurde über den Verlauf der Untersuchung informiert und erkläre mich mit der Durchführung der durch ein bildgebendes Verfahren gesteuerten Gelenkspunktion und der Applikation der Medikamente einverstanden.
