# Supplementary material for: The impact of peri-interventional factors on pain reduction in glenohumeral corticosteroid injections
Source: BMC Musculoskelet Disord. 2026 Mar 23;27:352. doi: 10.1186/s12891-026-09754-5 (PMC13107596; doi:10.1186/s12891-026-09754-5)
Supplement: Supplementary file 2 — Supplementary Material 2. [file 12891_2026_9754_MOESM2_ESM.docx]

# Supplementary Table S1. Sensitivity analysis excluding repeated injections.

| **Predictor** | **Coef (n=193)** | **95% CI** | **P-value** | **Coef (n=186)** | **95% CI** | **P-value** |
| --- | --- | --- | --- | --- | --- | --- |
| Intercept | 6.76 | 5.46 – 8.07 | <0.001 | 6.90 | 5.60 – 8.20 | <0.001 |
| Time: After Intervention | -1.97 | -2.45 – -1.48 | <0.001 | -1.92 | -2.41 – -1.43 | <0.001 |
| Time: One Week | -3.06 | -3.55 – -2.58 | <0.001 | -3.09 | -3.58 – -2.59 | <0.001 |
| Time: One Month | -2.95 | -3.44 – -2.47 | <0.001 | -2.98 | -3.47 – -2.49 | <0.001 |
| Treatment Group | 0.30 | -0.39 – 0.98 | 0.394 | 0.29 | -0.40 – 0.99 | 0.409 |
|  |  |  |  |  |  |  |

Seven patients underwent two separate injections and were originally recorded as independent observations. The left panel includes all observations (n = 193); the right panel excludes repeated injections (n = 186). Results remained materially unchanged.
